# A One-Year Survey of Norovirus in UK Oysters Collected at the Point of Sale

**DOI:** 10.1007/s12560-018-9338-4

**Published:** 2018-05-02

**Authors:** J. A. Lowther, N. E. Gustar, A. L. Powell, S. O’Brien, D. N. Lees

**Affiliations:** 1 Centre for Environment, Fisheries and Aquaculture Science, Weymouth, DT4 8UB UK; 2 Institute of Psychology, Health & Society, University of Liverpool, Liverpool, England, UK

**Keywords:** Norovirus, Oysters, qRT-PCR, Survey

## Abstract

Contamination of bivalve shellfish, particularly oysters, with norovirus is recognised as a food safety risk and a potential contributor to the overall burden of gastroenteritis in the community. The United Kingdom (UK) has comprehensive national baseline data on the prevalence, levels, and seasonality of norovirus in oysters in production areas resulting from a previous two-year study (2009–2011). However, previously, data on final product as sold to the consumer have been lacking. As part of a wider project to establish the overall burden of foodborne norovirus in the UK, this study aimed to address this data gap. A one-year survey of oysters collected from the point-of-sale to the consumer was carried out from March 2015 to March 2016. A total of 630 samples, originating in five different European Union Member States, were collected from 21 regions across the UK using a randomised sampling plan, and tested for norovirus using a method compliant with ISO 15216-1, in addition to *Escherichia coli* as the statutory indicator of hygiene status. As in the previous production area study, norovirus RNA was detected in a high proportion of samples (68.7%), with a strong winter seasonality noted. Some statistically significant differences in prevalences and levels in oysters from different countries were noted, with samples originating in the Netherlands showing lower prevalences and levels than those from either the UK or Ireland. Overall, levels detected in positive samples were considerably lower than seen previously. Investigation of potential contributing factors to this pattern of results was carried out. Application of normalisation factors to the data from the two studies based on both the numbers of norovirus illness reports received by national surveillance systems, and the national average environmental temperatures during the two study periods resulted in a much closer agreement between the two data sets, with the notably different numbers of illness reports making the major contribution to the differences observed in norovirus levels in oysters. The large majority of samples (76.5%) contained no detectable *E. coli*; however, in a small number of samples (2.4%) levels above the statutory end product standard (230 MPN/100 g) were detected. This study both revealed the high prevalence of norovirus RNA in oysters directly available to the UK consumer, despite the high level of compliance with the existing *E. coli*-based health standards, while also highlighting the difficulty in comparing the results of surveys carried out in different time periods, due to variability in risk factors.

**Electronic supplementary material:**

The online version of this article (10.1007/s12560-018-9338-4) contains supplementary material, which is available to authorized users.

## Introduction

Contamination of bivalve shellfish, particularly oysters, with norovirus is recognised as a food safety risk, with a considerable number of reports of outbreaks in the literature (reviewed in Bellou et al. 2013). In both the European Union and the United States, viral contamination in shellfish is regulated indirectly using enteric bacteria as an indicator of faecal pollution (Anonymous 2004, 2015b). However, this approach has been demonstrated to inadequately address the risk from human enteric viruses, with a number of viral outbreaks caused by batches compliant with the regulations (Chalmers and McMillan 1995; Le Guyader et al. 2008; Dore et al. 2010). Considerable progress has been made towards the development of detection methods for norovirus in molluscan shellfish and an ISO/CEN technical specification including such a method (ISO/TS 15216) was published in 2013 (Anonymous 2013), with a subsequent update to a fully validated standard in 2017 (Anonymous 2017). EU legislative texts foreshadow the adoption of virus controls in bivalve shellfish when the methods are sufficiently developed (Anonymous 2005) and the options for improvement of EU legislation to better address the virus risk have been actively discussed in recent years (EFSA Panel on Biological Hazards 2012). It has therefore become important to gain information about the application of the new methods, and the potential impact of possible legislative standards on bivalve shellfish production.

Amongst current European Union Member States, the United Kingdom (UK) has some of the most comprehensive national baseline data on the prevalence, levels and seasonality of norovirus in oysters resulting from a two-year study carried out on samples taken directly from production areas in 2009–2011. (Lowther et al. 2012b). However, until recently, data on final product as sold to the consumer in the UK have been lacking, with to the best of our knowledge the only published study having tested oysters from a single dispatch centre only (Lowther et al. 2010). As part of a wider project to establish the overall burden of foodborne norovirus in the UK (“NoVAS: Assessing the contribution made by the food chain to the burden of UK-acquired norovirus infection”—UK Food Standards Agency Project reference: FS101040), this study aimed to address this data gap, as well as to compare results for final product with those obtained in the previous production area survey.

## Materials and Methods

### Sampling Plan

The survey design was informed by a comprehensive practical evaluation of the purchase routes for oysters available to the UK consumer. This evaluation was undertaken by a specialist product retrieval company contracted to collect the survey samples (Stericycle ExpertSOLUTIONS, Reading, UK), through phone interviews with and physical visits to identified vendors. This market research was conducted in 21 selected cities/regions across the UK (selected to give a good geographical spread and including regions in each of the four constituent countries of England, Wales, Scotland and Northern Ireland). Vendors directly available to consumers of oysters were subdivided into the following types: supermarkets, fishmongers, restaurants, online sales and wholesalers. A total of 373 vendors were identified across the 21 areas.

A randomised sampling plan was drawn up aiming to obtain a total of 630 oyster samples over a 1-year period (16th March 2015–15th March 2016), with monthly targets of 26 samples in the truncated months of March 2015 and March 2016, and 53 or 52 samples alternatively for the months April 2015 to February 2016. For each month, the vendors targeted were selected randomly from a subset of the list of all 373 vendors with no weighting by region. Over the course of the survey, any shortfall in sample numbers collected in a given month was compensated by the addition of extra samples (selected at random from the same region for logistical regions) in the sampling schedule for the following month.

### Sample Collection

Samples (except online sales) were pre-ordered through direct contact with the vendor, then collected by sampling officers at the point-of-sale to the consumer. Samples sourced from online sales were ordered for delivery to the sampling officer. Within each vendor, samples were limited to native, Pacific, or other oyster species, sold as either ambient, chilled, or frozen. To avoid possible contamination by food handlers live animals in restaurants were obtained before shucking by restaurant staff. Cooked, pasteurised, smoked, or otherwise processed oysters were not sampled. Where multiple products or batches of the same product were available, one was picked at random by the sampler. A sample consisted of individual animals from the same batch (same origin and production date).

Given sufficient availability samples consisted of 25 oysters (with a minimum number of 12 oysters required for a valid sample). At the point of sampling, full sample details including date, time, vendor name and address, sample type, sample temperature at the point of sale (ambient, fresh, frozen), sample origin/identification mark (if available) were recorded by the sampling officer. A high-resolution digital photograph of the sample packaging and identification mark (if available) was taken. This information with accompanying photographs was then e-mailed to the Stericycle project co-ordinator for collation in a sample database.

Samples were packaged in temperature controlled Coleman food boxes with cool packs and despatched to the laboratory via overnight courier service accompanied by a sample submission form including a unique sample identifier, the date and time of collection, the storage temperature of the sample at the collection point and the date and time of despatch. Details of the vendor and the origin of the oysters were not included such that the sample testing was carried out blind.

Upon receipt at the laboratory, each sample was processed according to standard procedures. If the sample temperature on receipt was > 18 °C, fewer than 10 live animals were available, or the condition of the sample was otherwise unsatisfactory, samples were not tested, and replacement samples were collected. In addition, if the sample temperature on receipt was > 10 °C, fewer than 20 live animals were available, or a period of > 48 h had elapsed between sample collection and receipt at the laboratory, samples were analysed for norovirus only (not *E. coli*); under these circumstances replacement samples were not sought.

### Detection and Quantification of Norovirus

Oyster samples were tested for norovirus according to the draft international standard ISO 15216-1 (now published in Anonymous 2017).

#### Virus Extraction

For each sample, ten oysters were selected. The digestive tissues (stomach and digestive diverticula) of these oysters were excised, pooled, and then finely chopped using a razor blade. A 2-g subsample of chopped digestive tissues was transferred to a clean tube. 10 µl of mengo virus vMC0 tissue culture supernatant was added to the 2-g subsample as a within-sample virus/RNA extraction process control. Homogenates were prepared by adding 2 ml of a 100 μg/ml Proteinase K solution to the digestive tissues. This was then incubated at 37 °C with shaking at 320 rpm for a duration of 1 h, and subsequently incubated at 60 °C for a duration of 15 min. Finally, the sample was centrifuged at 3000×*g* for 5 min.; the volume of the soluble portion (homogenate) was measured and then retained for downstream testing and the pellet discarded. Homogenates were stored at 4 °C for up to one month prior to testing.

#### RNA Extraction

Total RNA was extracted from 500 µl of shellfish homogenate using a NucliSENS^®^ miniMAG extraction machine and NucliSENS^®^ magnetic extraction reagents (BioMerieux) following the manufacturer’s instructions (eluting in 100 µl elution buffer). A negative (water only) extraction control sample was also prepared and tested in parallel with each set of samples extracted. Eluted RNA was stored at − 20 °C until required.

#### One-Step qRT-PCR

For norovirus GI, QNIF4 and NV1LCR primers, and TM9 probe were used (da Silva et al. 2007; Hoehne and Schreier 2006; Svraka et al. 2007). For norovirus GII, QNIF2 and COG2R primers, and QNIFS probe were used (Kageyama et al. 2003, Loisy et al. 2005). For mengo virus, the mengo 110 and mengo 209 primers, and the mengo 147 probe were used (Costafreda et al. 2006). For both norovirus genogroup-specific assays, three aliquots of 5 μl sample or extraction control RNA was tested in 25 µl total volume with one-step reaction mix prepared using the RNA Ultrasense^®^ one-step qRT-PCR system (Invitrogen) (final concentrations of 1x Reaction Mix, 500 nM forward and 900 nM reverse primers, and 250 nM probe, plus 0.5 µl Rox and 1.25 µl Enzyme Mix per reaction). For mengo virus, two aliquots of 5 μl cDNA were used. Amplification was performed using the following cycling parameters: 55 °C for 60 min, 95 °C for 5 min, and then 45 cycles of 95 °C for 15 s, 60 °C for 1 min and 65 °C for 1 min on an Mx3005P real-time PCR machine (Stratagene). Wells containing nuclease free water and the above qRT-PCR reaction mixes were included on each plate as a negative control. Quantification used a log dilution series (range 1 × 10^5^ to 1 × 10^1^ copies/µl) of linear dsDNA molecules carrying the GI and GII target sequences and followed the principles outlined in ISO 15216-1 (Anonymous 2017). All samples were assessed for extraction efficiency by the comparison of sample Ct values for mengo virus with a standard curve generated from the process control material and for qRT-PCR inhibition using RNA external controls as described in ISO 15216-1 (Anonymous 2017). Samples were retested if extraction or qRT-PCR inhibition levels fell below 1% or above 75% respectively, where positive qRT-PCR controls indicated reagent failure, or for any positive sample where the negative extraction or PCR controls showed contamination. Quantitative results were not adjusted for losses during processing or RT-PCR inhibition.

### Detection and quantification of *E. coli*

Oyster samples were tested for *E. coli* according to ISO 16649-3 (Anonymous 2015a). Whole animal homogenates were prepared from the flesh and intravalvular fluid of 10 oysters and assayed using a most-probable-number (MPN) method. Results are expressed per 100 g of shellfish flesh and intravalvular fluid.

### Statistical Analysis

Relevant statistical analyses (Fisher’s exact test, Kruskal–Wallis test) were carried out using the Minitab software package. For statistical analysis and calculation of geometric means, positive results of < 100 copies/g (the limit of quantification of the assay) were scored at 50, and not detected samples were scored at 20 (half the limit of detection). Scores for GI and GII were combined prior to analysis. In this way, samples that were not detected for both genogroups scored 40 copies/g, and this figure should be considered a baseline for levels. Confidence intervals (95%) for datasets were calculated as the geometric mean ± 2*x* the geometric standard deviation; at the lower end, these are censored at 40 copies/g where the calculated value was less than this. Due to the large number of censored values in the dataset, non-parametric statistical tests were used throughout.

### Normalisation Factors

In order to compare the contribution of different risk factors to the results obtained in the current and previous studies, month-by-month normalisation factors were generated for norovirus illness (using data on illness reports in England and Wales provided by Public Health England) and environmental temperatures (using data on UK average air temperatures obtained from the UK Meteorological Office website—http://www.metoffice.gov.uk) as follows.

For illness reports, the normalisation factor *N*^i^ was determined as

$$ N^{\text{i}} = \frac{{I^{\text{a}} }}{{I^{\text{x}} }}, $$

where *I*^a^ is the average illness reports per day for the relevant calendar month in the period of the production area survey (May 2009–Apr 2011) and *I*^x^ average illness reports per day for the month in question, such that where illness reports for a given month were lower than the average for that calendar month in 2009–2011, the normalisation factor was > 1. For example, in April 2015, the average number of illness reports per day was 30.27, compared with the average for April during the production area survey of 34.05 reports per day. The normalisation factor for April 2015 was therefore 34.05 ÷ 30.27 = 1.12.

For temperatures, the normalisation factor *N*^t^ was determined as

$$ N^{\text{t}} = \frac{{20 - T^{\text{a}} }}{{20 - T^{\text{x}} }}, $$

where *T*^a^ is the long-term time series average temperature for the relevant calendar month (1981–2010) and *T*^x^ is the recorded monthly UK average temperature for the month in question, such that where the UK average air temperature for a given month was higher than the long-term average for that calendar month in 1981–2010, the normalisation factor was > 1. For example, in April 2015, the UK average temperature was 7.9 °C, compared with a long-term average for April of 7.4 °C. The normalisation factor for April 2015 was therefore (20 − 7.4) ÷ (20 − 7.9) = 1.04.

Normalisation factors calculated in this way were applied to the geometric mean norovirus levels recorded for each month of both the retail and production area surveys. For both surveys, an average level for each calendar month was calculated.

## Results and Discussion

### Norovirus Results

**Fig. 1:**
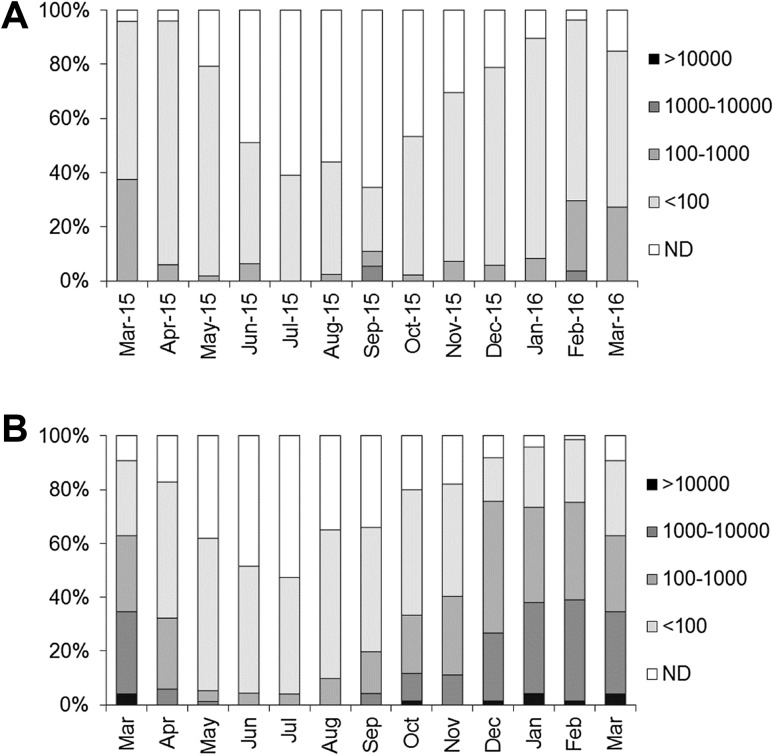
Monthly proportion of samples giving total norovirus results in different quantity brackets (copies/g) in the current (retail) survey and a previous production area survey. *ND* not detected. Results are for GI and GII combined; samples that were positive at levels of < 100 copies/g for both genogroups are included in the < 100 quantity bracket. **a** Results for the retail survey. **b** Results for the production area survey (Lowther et al. 2012b)—proportions calculated for each calendar month across the survey duration, March shown twice to allow comparison with the retail survey

All 630 samples subjected to testing passed quality control criteria for extraction efficiency and RT-PCR inhibition on initial or retesting. The average extraction efficiency obtained was 28.7% (range 1.1–99.6%), while the average RT-PCR inhibition was 14.0% (range 0–74.3%). Of the 630 samples, 433 (68.7%) were positive for norovirus RNA. Of these, 99 samples (15.7%) were positive for GI only, 88 (14.0%) were positive for GII only and 246 (39.0%) were positive for both GI and GII. A clear seasonality was observed with 79.7% of samples collected in the months October–March positive compared with 57.0% in the months April–September. This difference was found to be statistically significant (Fisher’s exact test; *p* < 0.0001). The highest and lowest monthly prevalences were recorded in February 2016 (96.3%) and September 2015 (34.6%), respectively (Fig. 1a).

Norovirus levels were also higher during the winter period with a geometric mean level of 87 copies/g (95% confidence interval 40–309 copies/g) in the months October–March compared with 65 copies/g (95% confidence interval 40–202 copies/g) in samples collected from April to September. This difference was found to be statistically significant using the Kruskal–Wallis test (*p* < 0.001). The highest levels recorded in individual samples were 586 copies/g for GI and 1802 copies/g for GII; however, in the majority of samples testing positive (85.9%), the levels recorded were below the limit of quantification of the assay (100 copies/g) for both norovirus GI and GII. In total, 61 samples produced results of > 100 copies/g for one or both genogroups, representing 14.1% of positive samples, and 9.7% of total samples. Of these 61 samples, 7 produced results of > 100 copies/g for both genogroups, 2 for GI only and 52 for GII only. The highest monthly incidence of samples giving results > 100 copies/g was March 2015 (37.5%). Over the course of the survey, 5 samples (0.8% of total samples) produced results for GI and GII combined of > 1000 copies/g; three of these samples were collected in September 2015, and 2 in February 2016.

### Comparison of Oysters Originating in Different Countries

For 492 samples (78.1% of the total), the dispatch centre from which the oysters originated could be identified as a result of information collected by the sampling officer (this identification was supported by a photograph of the identification mark or other identifying labels/packaging in 378 cases). Oysters originated from 33 different dispatch centres in 5 different EU Member States. Of the 492 samples with identified dispatch centres, 434 samples (88.2%) originated in the UK, 29 (5.9%) from the Netherlands, 25 (5.1%) from Ireland, 3 (0.6%) from France and 1 (0.2%) from Spain. Prevalences of norovirus detection and geometric mean levels of norovirus for samples originating in different EU Member States are shown in Table 1.

**Table 1:** Norovirus results by country of origin

Country of origin	Number of samples	Norovirus results	
		Prevalence (% positive) (%)	Geometric mean (copies/g; 95% confidence interval in parentheses)
UK	434	71.7	78 (40–277)
Netherlands	29	31.0	49 (40–91)
Ireland	25	84.0	69 (40–120)
France	3	33.3	48 (40–92)
Spain	1	100.0	275 (n/a)

Overall prevalence and levels of norovirus were lower in samples originating outside the UK (55.2% of samples positive, geometric mean of 58 copies/g, 95% confidence interval 40–129 copies/g) than in samples from the UK (71.7% positive, geometric mean of 78 copies/g, 95% confidence interval 40–277 copies/g). These differences were found to be statistically significant (Fisher’s exact test, *p* = 0.0144; Kruskal–Wallis test, *p* < 0.001). Further subdivision of non-UK samples to enable country-by-country analysis showed that for oysters from the Netherlands both prevalence and levels were significantly lower than for the UK (Fisher’s exact test, *p* < 0.0001; Kruskal–Wallis test, *p* < 0.0001). Prevalence and levels for oysters from the Republic of Ireland were not significantly different from those for the UK, but were significantly higher than those for the Netherlands (Fisher’s exact test, *p* = 0.0001; Kruskal–Wallis test, *p* = 0.0081). No apparent seasonal bias in collection dates for samples from the three countries were found to explain these differences (no significant difference was found between the proportions of samples collected during the winter months October–March using Fisher’s exact test). Statistical analysis of norovirus results for samples from France and Spain was not carried out due to the small number of samples.

### Comparison with the Production Area Study

The prevalence of norovirus RNA in oyster samples recorded in this survey (the “retail survey”; 68.7%) was similar but slightly lower than that found in a previous two-year survey (2009–2011) of oysters from UK production areas (the “production area survey” 76.2%) (Lowther et al. 2012b). In addition, a similar seasonality with increased prevalences and levels in the winter months was noted in both surveys. However, the overall levels of norovirus recorded in the retail survey were considerably lower than in the production area survey. In the latter, 36.5% of total samples contained levels > 100 copies/g (the limit of quantification of the assay) for one or both norovirus genogroups, combined levels of > 1000 copies/g were found in 14.6% of samples, and combined levels of > 10,000 copies/g were found in 1.1% of samples. Geometric means for all results were 76 copies/g (95% confidence interval 40–261 copies/g) and 159 copies/g (95% confidence interval 40–2964 copies/g) for the retail and production area surveys, respectively. This difference was found to be statistically significant (Kruskal–Wallis test; *p* < 0.001).

Possible underlying causes for this pattern of results include:-

1. Risk reduction measures by Food Business Operators including e.g. use of enhanced depuration conditions or use of norovirus testing to inform decisions on choice of supply for processing and marketing
2. Representativeness of samples: It is possible that the production area survey was not representative of the volumes of oysters placed on the UK market as the selection of sites for the production area survey was meant to provide a representative selection of production areas with different risk profiles and a good geographical spread, but not to represent production volumes or market share.
3. Variation in norovirus shedding rates in the community: Sewage treatment is known to only reduce norovirus by a limited extent (Campos and Lees 2014). Consequently, a key factor influencing norovirus contamination in filter-feeding shellfish impacted by sewage discharges will be the degree of virus infection, and hence the degree of virus shedding in faeces, in the population contributing to the sewage inputs. During this study, unusually low levels of norovirus were observed in the community in England and Wales during the winter of 2015–2016, particularly during the months November to January, compared with unusually high levels during the winters of 2009–2010 and 2010–2011 (Supplementary Figure S1, data provided by Public Health England, equivalent data for other parts of the UK are not available).
4. Variation in environmental temperatures: Shellfish are poikilothermic (Gosling 2008), and their metabolic rate, and hence the degree of contaminant uptake and removal, is significantly influenced by the temperature of their environment. In this study, environmental temperatures in the UK were unusually high during the winter of 2015–2016, particularly during the months November to January, compared with unusually low temperatures during the production area study winters of 2009–2010 and 2010–2011 (Supplementary Figure S2; data obtained from the UK Meteorological Office website—http://www.metoffice.gov.uk).

Of the above factors potentially influencing the variation seen between contamination levels in the production area study and in this study, it was only possible to perform further analysis on the impact of general population shedding rates and environmental temperatures due to the unavailability of data relevant to the other factors. To further investigate these possible contributing elements, month-by-month normalisation factors were determined using the PHE data on illness reports in England and Wales (treating these figures as a proxy for community levels as a whole) and Met Office data on UK national average monthly air temperatures (treating these figures as a proxy for environmental temperatures as a whole—equivalent national average seawater temperatures are not available) as described in materials and methods.

**Fig. 2:**
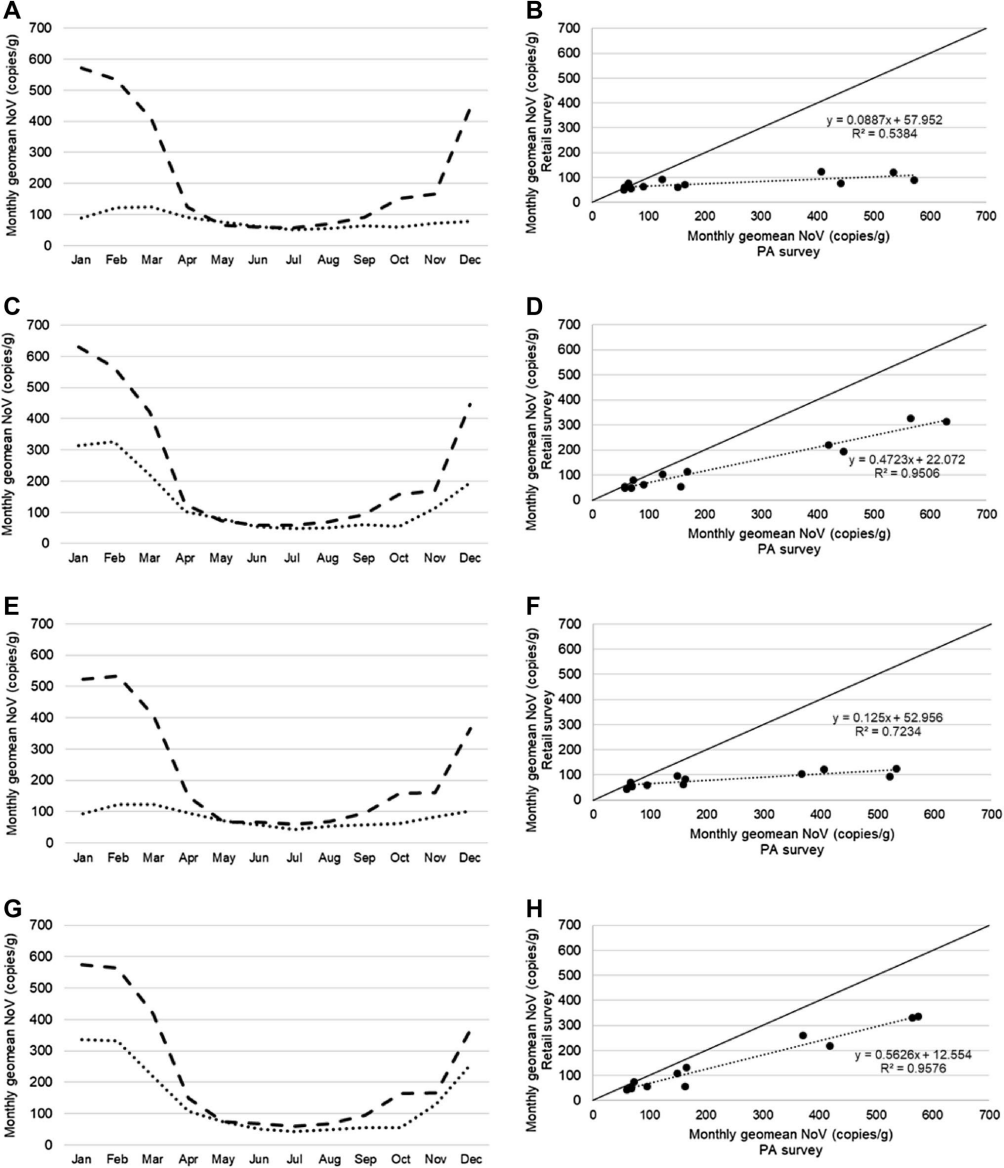
Application of normalisation factors to monthly geometric mean norovirus levels obtained during the retail and production area surveys. **a**, **c**, **e**, **f** comparison of monthly geomean levels for the retail (dashed lines) and production area (dotted lines) surveys. **b**, **d**, **f**, **h**; correlation between geometric mean norovirus levels for each calendar month obtained during the two surveys. Lines of equality (solid) and best fit (dotted and labelled with associated equation and *r*^2^ values) are shown. **a**, **b** No normalisation applied. **c**, **d** Normalisation factors derived from illness reports applied. **e**, **f** Normalisation factors derived from average temperatures applied. **g**, **h** Normalisation factors derived from illness reports and average temperatures applied

Application of the normalisation factors based on illness reports resulted in a notable improvement in correspondence in results by calendar month between the two surveys (see Fig. 2). Geometric mean levels for each month in the two surveys are plotted against each other in Fig. 2b, d, f, h alongside lines of best fit and equality; for data normalised according to illness reports (Fig. 2c, d), the slope of the line of best fit (0.4723) is considerably closer to equality and the correlation is considerably closer to total (*r*^2^ = 0.9506) than for non-normalised data (Fig. 2a, b; slope = 0.0887 and *r*^2^ = 0.5384).

Application of the normalisation factors for temperature in isolation yielded only a modest improvement in agreement between the results of the two studies (Fig. 2e, f). However, application of both the illness and temperature-based normalisation factors in combination produced the best line of best fit overall (Fig. 2g, h; slope = 0.5626 and *r*^2^ = 0.9576).

This analysis indicates that much of the difference in the norovirus levels between the retail and production area surveys can be attributed to the different levels of norovirus in the community between the two study periods, with some portion of the remaining difference explained by the differing temperatures, particularly during the early part of winter. Nevertheless, even normalising using these factors together results in levels in the retail study on average ~ 56% as high as during the production area survey, suggesting other factors as discussed above also contributed to the different pattern of results.

### *E. coli* Results

Out of 630 samples received, *E. coli* analysis was carried out in 452 cases (71.7%). For the remaining samples, *E. coli* testing was not carried out primarily due to insufficient live animals in the sample to conduct this test in addition to norovirus analysis (< 20), or elevated temperatures on arrival (> 10 °C).

Of the samples tested for *E. coli*, the bacterium was not detected (< 18 MPN/100 g) in 346 cases (76.5%). In 11 samples (2.4%), levels in excess of the EU legal end product standard (230 MPN/100 g; Anonymous 2005) were detected. In these cases, the UK Food Standards Agency as the Competent Authority was informed on the same working day that the result became available. All 11 of these samples were collected between March and September 2015, with the highest monthly incidence of five samples > 230 MPN/100 g in July 2015, representing 15.2% of the samples collected in that month. In one sample, a level in excess of the upper limit of quantification of the *E. coli* assay (> 18,000 MPN/100 g) was recorded from a sample collected on 15 July 2015.

In comparison with the production area survey, levels of *E. coli* recorded in this study were very low. No *E. coli* was detected in the majority of the samples, while results over the A classification and end product standard were rare. In the production area survey by contrast, *E. coli* proportions were 14.3% undetected and 40.0% > 230 MPN/100 g. Although other factors may have contributed, this difference is likely to be largely the result of the well-established high efficacy of standard depuration conditions for the removal of *E. coli* bacteria (Doré and Lees 1995). Since the removal of *E. coli* is a good proxy for other bacterial pathogens derived from sewage contamination (Lees 2000), this demonstrates the contribution to public health of the classification and depuration regulations for protection from bacterial illness. This finding is supported by the low numbers of bacterial infections associated with consumption of oysters in the UK (Lees 2000).

The small number of results of > 230 MPN/100 g, including one result of > 18,000 MPN/100 g, indicates that despite the high level of adherence to the legal standards, problems can nevertheless occur. The root cause of the high *E. coli* levels detected in some samples could not be investigated, but could conceivably be linked to problems post-harvest, during transportation, or at the point-of-sale.

## Conclusion

The survey described here is the first systematic study of norovirus in oysters collected at the point-of-sale in the UK. Norovirus RNA was detected in 68.7% of samples tested, comparable with the prevalence found in a previous survey carried out using the same methods on oysters from UK production areas (76.2%; Lowther et al. 2012b). The prevalence described here is considerably higher than recorded in surveys of norovirus in bivalve shellfish collected at the point-of-sale in some other countries, for example the United States (3.9%; Woods and Burkhardt 2010), France (9%; Schaeffer et al. 2013) and Thailand (12.3%: Kittigul et al. 2016); however, comparatively frequent detection of norovirus has been reported in shellfish from production areas in Ireland (37.1%; Flannery et al. 2009), Italy (51.5%, Suffredini et al. 2014) and Spain (52.4%; Polo et al. 2015). Although the majority of samples were found to be positive, levels exceeding 100 norovirus copies/g were found in only a relatively small percentage of samples (9.7%). The relationship between levels of norovirus as detected by PCR and human health risks is complex (EFSA Panel on Biological Hazards 2012); however in an analysis of outbreak-related oyster samples carried out in this laboratory (Lowther et al. 2012a), an association between increased norovirus levels and increased likelihood of norovirus-type illness was observed, with no outbreak-related sample recording levels below 152 copies/g. The human health consequences of the large proportion of positive samples in this survey are therefore not certain.

The majority of oyster samples tested originated from dispatch centres in the UK (88.2% of samples where the dispatch centre could be identified), with the remainder originating in other countries in Western Europe. Systematic comparison of prevalences and levels of norovirus in oysters from different countries was complicated by the low numbers of samples from each exporter country; however oysters from the Netherlands showed significantly lower levels and prevalences than oysters from both the UK and Ireland. There is some evidence that oyster growing waters in the Netherlands are impacted by lower levels of faecal pollution; six out of seven (86%) oyster production areas in the country are at the time of writing classified A (Netherlands National Reference Laboratory for monitoring bacteriological and viral contamination of bivalve molluscs; personal communication), the cleanest status based on *E. coli* monitoring results according to EU legislation (Anonymous 2004). By contrast, in the UK, 37% of oyster production areas are wholly or partially classified A, either permanently or for part of the year (UK National Reference Laboratory for monitoring bacteriological and viral contamination of bivalve molluscs; personal communication).

During the year of the survey, some significant potential risk factors were low compared with the previous study on oysters from UK production areas (Lowther et al. 2012b). The number of norovirus cases in the general population and hence the likely extent of virus shedding into shellfish production areas was considerably lower than previously, and environmental temperatures during the winter were higher. The datasets used to quantify these risk factors had some limitations; air temperatures were used as an indicator of overall environmental temperatures, rather than directly using seawater temperatures (no national average seawater temperature data is available). In addition, for both factors, data collected in the UK were extrapolated to normalise results based on all samples collected during the retail survey, including those originating outside the UK. However, 430 out of 432 samples (99.6%) where origin data existed either originated in the UK, or in bodies of water abutting UK territorial waters (the Irish Sea, the English Channel and the North Sea), while the illness data used broadly reflects global trends in norovirus infections. The two winter periods in which illness levels in the dataset used were highest (2009–2010 and 2012–2013) both followed directly on from the emergence of a global pandemic strain; New Orleans 2009 (Vega et al. 2011) and Sydney 2012 (van Beek et al. 2013), respectively. For these reasons, we therefore considered that use of these suboptimal datasets for determination of normalisation factors was unlikely to confound the analysis we carried out.

This analysis offers some insights into the contribution of these two factors to the pattern of results observed in the different surveys, and highlights the difficulty of comparing results from surveys carried out in different time periods, or of treating the results of a short survey as completely indicative of the long-term characteristics of the surveyed area. It is however possible that some of the differences observed were down to inherent differences between production area and retail-ready oysters. For example, Food Business Operator risk management interventions (such as virus testing, or selection of product from cleaner areas) may have contributed to the low virus levels seen in this study. Direct comparison, within the same time period, of levels in production areas with those seen in retail-ready oysters would assist assessment of the contribution made by producer practices. An ongoing EU-wide survey of norovirus in oysters from both production areas and dispatch centres, organised by the European Food Safety Authority (EFSA 2012) may help to illuminate this issue.

The very high proportion of samples compliant with the *E. coli* end product standard (97.6%) in this study indicates the good compliance with current regulatory requirements in the UK oyster supply chain and the consequential probable low risk from bacterial pathogens such as *Salmonella*. However, the contrast between the results for norovirus and *E. coli* again illustrates the limitations of this organism as an indicator of viral risk in shellfish, particularly in depurated animals. Shellfish-related outbreaks of norovirus have often been linked to batches compliant with the end product standard for *E. coli* (Chalmers and McMillan 1995; Le Guyader et al. 2008; Dore et al. 2010). On the other hand, we have previously observed a site-by-site correlation between *E. coli* and norovirus levels in oysters from UK production areas (Lowther et al. 2012b).

In summary, although illuminating the difficulties with comparing data collected in different periods, this study provides a comprehensive and systematic assessment of the extent of both norovirus and *E. coli* contamination in oysters available to the UK consumer. These data can assist risk managers both in the UK and beyond to manage the health risks posed by norovirus in oysters, and as part of the wider NoVAS project will contribute to an assessment of the contribution of the food chain to norovirus infections in the UK.

## Electronic supplementary material

Below is the link to the electronic supplementary material.

Supplementary material 1 (DOCX 42 kb)
