# Supplementary material for: A One-Year Survey of Norovirus in UK Oysters Collected at the Point of Sale
Source: Food Environ Virol. 2018 May 2;10(3):278–87. doi: 10.1007/s12560-018-9338-4 (PMC6096945; doi:10.1007/s12560-018-9338-4)

**Supplementary Figure S-1** Monthly average number of community norovirus illness reports per day in England and Wales during the study periods of the retail and production surveys (data provided by Public Health England).


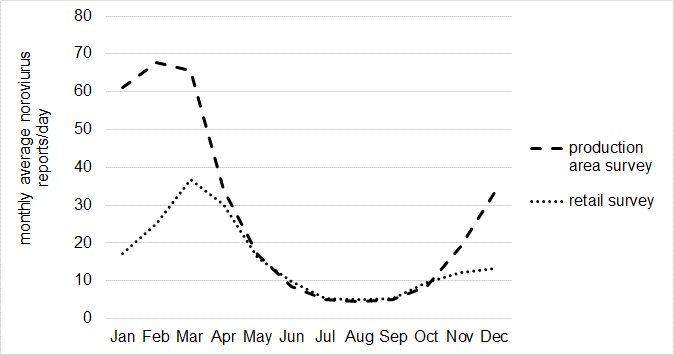


**Supplementary Figure S-2** Monthly average temperatures in the UK during the during the study periods of the retail and production surveys, compared with the long term average (data obtained from the UK Metereological Office website - <http://www.metoffice.gov.uk>).


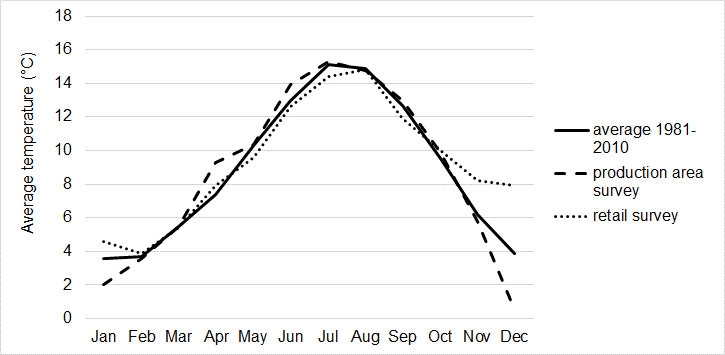

Supplement: Supplementary file 1 — Supplementary material 1 (DOCX 42 kb) [file 12560_2018_9338_MOESM1_ESM.docx]
